# Fluid Dynamics Experiments for Planetary Interiors

**DOI:** 10.1007/s10712-021-09681-1

**Published:** 2021-12-10

**Authors:** Michael Le Bars, Ankit Barik, Fabian Burmann, Daniel P. Lathrop, Jerome Noir, Nathanael Schaeffer, Santiago A. Triana

**Affiliations:** 1 CNRS, Aix Marseille Univ, Centrale Marseille, IRPHE UMR 7342, 13013 Marseille, France; 2 Johns Hopkins University, 3400 N. Charles Street, Baltimore, 21210 USA; 3 Institute of Geophysics, ETH Zurich, Sonnegstrasse 5, 8092 Zurich, Switzerland; 4 University of Maryland, College Park, MD 20742-4111 USA; 5 Univ. Grenoble Alpes, CNRS, ISTerre, 38000 Grenoble, France; 6 The Royal Observatory of Belgium, Avenue Circulaire 3, 1180 Uccle, Belgium

**Keywords:** Planetary cores, Subsurface oceans, Rotational fluid dynamics, Waves, Instabilities, Turbulence

## Abstract

Understanding fluid flows in planetary cores and subsurface oceans, as well as their signatures in available observational data (gravity, magnetism, rotation, etc.), is a tremendous interdisciplinary challenge. In particular, it requires understanding the fundamental fluid dynamics involving turbulence and rotation at typical scales well beyond our day-to-day experience. To do so, laboratory experiments are fully complementary to numerical simulations, especially in systematically exploring extreme flow regimes for long duration. In this review article, we present some illustrative examples where experimental approaches, complemented by theoretical and numerical studies, have been key for a better understanding of planetary interior flows driven by some type of mechanical forcing. We successively address the dynamics of flows driven by precession, by libration, by differential rotation, and by boundary topography.

## **Article Highlights**

- Laboratory experiments are used to investigate the rotational dynamics of planetary internal fluid layers, including cores and subsurface oceans.
- Various types of waves, instabilities, and turbulence are quantitatively described.
- Consequences regarding magnetic field generation and kinetic energy dissipation are addressed.

## Introduction

Numerous planetary bodies have or had a global, internal fluid layer, such as a liquid iron-rich core in the deep interior of terrestrial planets and moons, or a salty water ocean below the solid surface of icy satellites. Understanding the flows taking place in these spherical shell envelopes remains a tremendous interdisciplinary challenge, despite more than one century of intense research. Beyond the challenge in fundamental fluid dynamics to understand these complex motions involving turbulence, rotation, and buoyancy effects at typical spatial and temporal scales well beyond our day-to-day experience, a global knowledge of the involved processes is fundamental to a better understanding of the global dynamics of planets. Indeed, turbulent flows in cores and oceans significantly influence the planets thermal and orbital evolution, because of heat advection, viscous dissipation, and coupling with the overlying/underlying solid shells. Also, motions in conducting fluids are the main mechanism for generating planetary magnetic fields (Larmor 1919), a possible ingredient for planetary habitability. Generally speaking, and even though the deeper interior dynamics is not directly observable, gravity data, magnetic field, and the rotation state of a planet are influenced by ongoing flows and hence offer indirect clues for their understanding.

The main obstacle to quantitative modeling and understanding of planetary hidden flows stands in the extreme character of the involved physical dimensionless parameters, which translates to highly turbulent regimes implying an extremely wide range of time and length scales. For instance, the relative importance of viscous and Coriolis forces is measured by the Ekman number

1 $$\begin{aligned} Ek= \frac{\nu }{\varOmega R^2}, \end{aligned}$$

where $\nu$ is the fluid viscosity, $\varOmega$ the planet rotation rate, and *R* the typical depth of the considered fluid layer. Another relevant dimensionless parameter is the Rossby number which compares the flow nonlinearity and the Coriolis force

2 $$\begin{aligned} Ro=\frac{U}{\varOmega R}, \end{aligned}$$

where *U* is the typical large-scale velocity of the flow. As will be seen below, *Ro* is also often considered as a dimensionless measure of the amplitude of the flow source. The ratio of the Rossby and Ekman numbers defines the Reynolds number

3 $$\begin{aligned} Re=\frac{UR}{\nu }, \end{aligned}$$

which schematically measures the turbulence intensity of the flow. Earth’s core, for instance, is a very turbulent, fast rotator with $Ek \simeq 10^{-15}$ and $Re \simeq 10^8$, whose nonlinear dynamics is strongly constrained by rotation as shown by $Ro \simeq 10^{-7}$. Those values are clearly out of reach of all our available investigation tools in the laboratory. Direct numerical simulations performed with the present day, most powerful, computational methods reach $Ek \simeq 10^{-7}$ and $Re \simeq 5 \times 10^3$ (Schaeffer et al. 2017), but a single computational run takes months for a few turnover times. Standard numerical simulations remain more than one order of magnitude below those record numbers. Some numerical tricks allow for a larger exploration of the parameter space, for instance by parameterizing the smallest scales of the dynamics (i.e., the so-called large eddy simulations (Aubert et al. 2017), well known in engineering sciences), or by considering relevant asymptotic developments (e.g., the quasi-geostrophic limit $Ro \rightarrow 0$, see Calkins et al. 2015; Guervilly et al. 2019); but those approximated models always require solid validations and careful interpretation.

As a result, most relevant studies of planetary fluid dynamics rely on the general principle of dynamical similitude and scaling laws, sustained by asymptotic theory: Because it is impossible to reproduce in a model the exact parameters of a planetary flow, the effort is focused on reaching the same dynamical regime, with the correct hierarchy of forces, i.e., $Ek \ll 1$, $Re \gg 1$, and $Ro \ll 1$. A systematic exploration of the parameter space then allows deriving scaling laws that are extrapolated toward planetary scales and challenged against available data (e.g., Christensen et al. 2009). In this approach, laboratory experiments are particularly useful. Indeed, a now reasonably affordable experimental setup in water, with $\varOmega$ equal to 1 rotation per second, $R=0.5$ m and a typical velocity $U=0.1$ m/s, gives $Ek \simeq 6\times 10^{-7}$, $Re \simeq 6\times 10^4$ and $Ro \simeq 3\times 10^{-2}$, which is still far from planetary values, but nevertheless closer than standard simulations, and more turbulent. Besides, once they are settled, experiments allow for the systematic exploration of a large parameter space, using long data acquisition. In addition to various types of probes and sensors locally measuring the pressure, temperature, magnetic field, etc., at a high acquisition rate (e.g., Zimmerman et al. 2014), non-intrusive techniques like laser Doppler anemometry (LDA, e.g., Noir et al. 2012), ultrasonic Doppler velocimetry (UDV, e.g., Noir et al. 2001), and particle image velocimetry (PIV, e.g., Le Reun et al. 2019) are now commonly available, giving detailed access to the velocity along a line and in a chosen plane, respectively. The main drawback of the experimental approach stands in the limitations of accessible geometries and physics. In particular, spherical geometries with radial gravity are accessible only partially using the centrifugal gravity (Busse and Carrigan 1976) or in complex, microgravity setups (Zaussinger et al. 2018); and to date, only few dynamo experiments have been successful, and always in constrained flows (Stieglitz and Müller 2001; Gailitis et al. 2001) or with specific boundary conditions (Berhanu et al. 2010). For successfully tackling planetary flow regimes and their consequences, both numerical and experimental approaches are thus fully complementary.

The present article reviews four selected configurations where experimental studies have significantly contributed to understanding key aspects of planetary interior flows. We successively address below the dynamics driven by precession (Sect. 2), by libration (Sect. 3), by differential rotation (Sect. 4), and by boundary topography (Sect. 5), in planetary relevant contexts. All these flows differ by their source of forcing, but they all take place in a rapidly rotating environment and accordingly share some generic physical ingredients. First, accounting for the small values of the Ekman number, viscous effects are mainly concentrated into thin layers close to the rigid boundaries called Ekman layers, whose thickness scales like $\sqrt{\nu /\varOmega } = R Ek^{1/2}$. Additional thin viscous layers can also appear in the bulk of the fluid to connect differentially rotating domains (Stewartson 1966): they are then called Stewartson layers. But the bulk of the fluid can to a large extent be considered as inviscid. Second, rotating flows carry a specific type of transverse waves sustained by the Coriolis force. These so-called inertial waves have a peculiar dispersion relation where their wave vector makes an angle $\theta$ with the rotation axis that depends only on their frequency $\omega$: $\cos (\theta )={\omega }/{2\varOmega }$. As a result, their frequency is bounded by $|\omega | \le 2\varOmega$ (Greenspan 1968). Inertial wave reflections at rigid boundaries conserve the angle $\theta$. If the reflected wave is parallel to the solid wall, the energy is completely absorbed in the Ekman boundary layer leading to an increased thickness, often referred to as an eruption and an enhanced Ekman pumping. In closed containers, the reflections result in a discrete set of closed trajectories, attractors or inertial modes, that might be resonantly excited. And third generic behavior shared by all rotating flows: In the low-frequency limit, the dynamics is dominated by the geostrophic force balance between the Coriolis acceleration and the pressure gradient, leading to flow structures elongated in the direction parallel to the rotation axis. These quasi-geostrophic flows are omnipresent in geophysical contexts in the form of cylindrical shear, isolated Taylor columns, and Rossby waves, as observed in rapidly rotating experiments and numerical simulations.

These three main generic features of rotating flows form the basis for their surprising and rich dynamics, which will be now illustrated through our four representative examples.

## Flows Driven by Precession

**Fig. 1:**
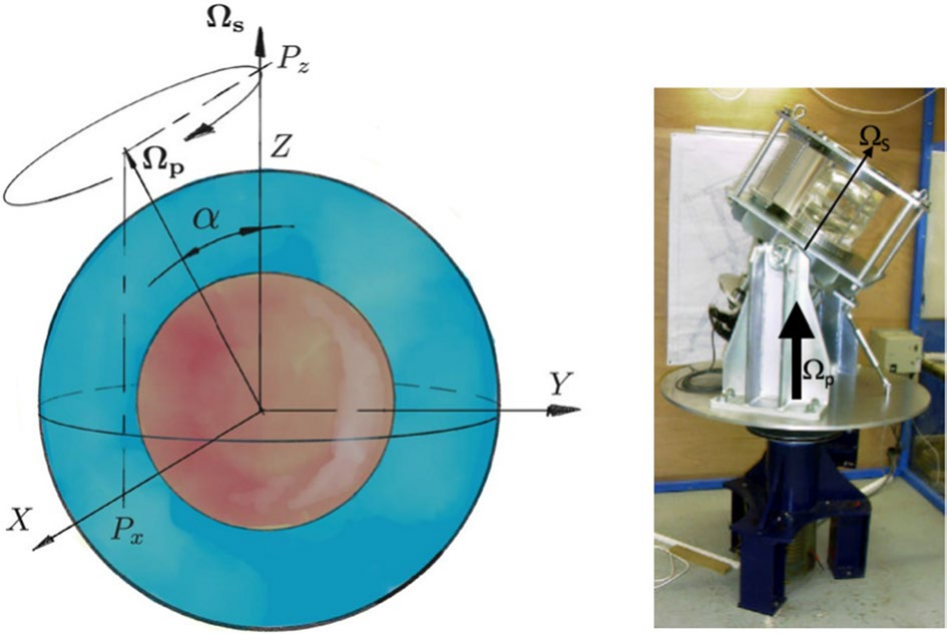
Schematic view of a precessing body, and experimental arrangement of Noir et al. (2001): a spheroidal cavity is filled with water and rotates along the spin axis ${\varvec{\varOmega }_\mathbf{S}}$ tilted versus the precession axis ${\varvec{\varOmega }_\mathbf{P}}$, set in the experiment by the slowly rotating turntable

By the term *precession*, we designate a motion analogous to the one of a gyroscope: It rotates rapidly along its spin axis, while the spin axis itself slowly rotates—precesses—around the precession axis (see Fig. 1). For planets and moons, the gyroscopic torque balances torques arising from the tidal forces exerted by their main orbital partners acting on their non-spherically symmetric solid mantle. On Earth today, the lunisolar gravitational torque produces a precession of its spin axis with a period of about 26,000 years around the normal to the ecliptic plane with an apex angle of $\alpha =23.5^\circ$. In addition to this angle $\alpha$, precession is characterized by the dimensionless Poincaré number *Po*, which measures the ratio of the rotation period to the precession period $Po=T_s/T_p$, or $Po=\varOmega _p/\varOmega _s$ using the precession rate $\varOmega _p$ and the spin rate $\varOmega _s$. $\alpha$ and *Po* are specific parameters to precession forcing, but they can be used to define a Rossby number characteristic of the forcing amplitude as $Ro = Po \sin {\alpha }$. For the Earth, $Po \simeq 10^{-7}$ and $Ro \simeq 4\times 10^{-8}$, we thus expect the flow to be strongly influenced by the rotation.

To understand the fundamental physics of a fluid gyroscope, let us first consider the case of an inviscid fluid in a spherical shell. In such a case, the liquid decouples from the boundaries and remains in a steady rotation while the surrounding shell precesses. Reintroducing the viscosity, still in a spherical cavity, the viscous stress at the solid–liquid interface will communicate the precessional motion of the shell to the liquid. This causes the spin axis of the fluid to also precess at the same rate but with a different axis: The gyroscopic torque is balanced by the viscous torque. Hence, the rotation axis of the fluid appears tilted compared to the one of the solids. The viscous coupling torque is proportional to ${Ek}^{1/2}$, a dimensionless measure of the strength of viscous forces typically of order $10^{-7}$ for planets [see definition of the Ekman number in (1)]. Departure from a purely spherical boundary will induce an additional pressure (also called topographic) torque that will reinforce the coupling between the fluid and the solid, further reducing the tilt. For planets, the non-sphericity is due primarily to planetary rapid rotation resulting in an oblate spheroidal shape—a sphere flattened at the poles. For Earth, the polar flattening $\eta =(a-c)/a$, with *a* and *c* the equatorial and polar radii, respectively, is on the order of 1/300 at the surface and $\sim 1/400$ at the core–mantle boundary (CMB), but these values can vary significantly for different celestial objects in the solar system ranging from 1/10 for Saturn to 1/900 for the Moon. The pressure torque resulting from the deformed CMB acting on the fluid core is proportional to the polar flattening $\eta$ (Noir et al. 2003). In planetary settings where $1/10 \le \eta \le 1/900$, the pressure torque is thus expected to largely dominate the viscous laminar torque.

Gravitational coupling with an orbital companion can further distort the spheroidal shape into a triaxial ellipsoid. This is most pronounced for tidally locked celestial objects, i.e., objects that always show the same face to their orbital partner, for example the Earth’s Moon. The pressure torque in this case depends on a combination of the equatorial and polar ellipticities, yet still dominating the laminar viscous torque in planetary cores.

It is thus legitimate to first consider the inviscid response of a fluid spheroidal cavity as in the pioneer work of Poincaré, Sloudsky, and Hough at the end of the nineteenth century, assuming a quasi-solid body rotation response (Poincaré 1910; Sloudsky 1895; Hough 1895). Later, Busse (1968) reintroduced the viscous torque deriving the first complete and self-consistent predictive model of the fluid rotational response. A fundamental outcome from these studies is the existence of an abrupt increase in the tilt of the core rotation axis as the precessional rate approaches the frequency of the so-called Poincaré mode, also referred to as the spin-over mode, or the free core nutation (FCN) by astronomers. FCN is actually the simplest inertial mode of the fluid core, resembling a solid body rotation around an equatorial axis (see detail in, e.g., Le Bars et al. 2015). Its frequency depends essentially on the polar ellipticity. For precession periods much larger than the FCN, the core is strongly coupled with the mantle, e.g., on Earth the tilt of the core rotation axis with respect to that of the mantle is of the order of $10^{-6}$ degree. Conversely for precession periods much shorter than the FCN, the core decouples from the mantle, leading to large differential rotation, as for the Earth’s Moon for which the 18.6 yrs period precession decouples the lunar core from the lunar mantle with a tilt of the order of $1.5^\circ$ (Cébron et al. 2019).

When large enough, the differential motion between the fluid and the solid shell can lead to turbulent flows providing an efficient mechanism to dissipate energy and possibly sustain electrical currents. Those effects can leave observable signatures such as a self-generated magnetic field or influence the planet’s orbital dynamics (Tilgner 2005; Lin et al. 2016; Reddy et al. 2018; Cébron et al. 2019). Understanding these phenomenons is key to probing planetary interiors with remote observations, yet these turbulent flows are out of reach of numerical investigations and we must turn to experiments to investigate them in connection with theoretical investigations.

There are not many precession experiments, arguably because they are difficult to perform. In order to reach low Ekman numbers that are relevant for geophysical applications ($Ek=10^{-15}$ in the Earth’s core), rapid rotation is required in laboratory experiments. In order to enforce precession, the setup uses two independent motors, one mounted on top of the other. A typical setup is presented in Fig. 1, and Table 1 lists the characteristics of the main precession setups. For comparison, Earth’s core values and the parameter range accessible by numerical simulations are also given. Interestingly, any rotating cavity around a fixed axis is subject to the 24 hours rotation of the Earth, which plays the role of the precession turntable. Although very small, this effect has been observed in dedicated experiments (Vanyo and Dunn 2000; Boisson et al. 2012; Triana et al. 2012).

**Table 1:** Comparison of selected experimental studies of precession of liquid-filled spheroidal or spherical containers, along with the Earth’s core, state-of-the-art numerical simulations, and the future Dresdyn precessing cylinder experiment

Reference	Ekman number	Poincaré number	Angle	Ellipticity
Earth’s core	$10^{-15}$	$10^{-7}$	$23.5^\circ$	0.0025
Malkus (1968)	$\ge 2 \times 10^{-6}$	0.003–0.08	$30^\circ$, $96^\circ$	0.04, 0.1
Vanyo et al. (1995)	$7.5 \times 10^{-7}$	$5\times 10^{-4}$–0.0575	$23.5^\circ$	0.01
Vanyo and Dunn (2000)	$\ge 2 \times 10^{-7}$	$10^{-6}$–$5 \times 10^{-4}$	$23.27^\circ$	0.0025
Noir et al. (2001)	$3\times 10^{-6}$	$5\times 10^{-4}$–0.05	$20^\circ$	0.04
Cébron et al. (2010), Nobili et al. (2021)	$3\times 10^{-6}$–$2\times 10^{-4}$	0.01–0.25	$5^\circ$, $15^\circ$	0.15
Goto et al. (2014), Horimoto et al. (2020)	$1.25\times 10^{-5}$–$2\times 10^{-4}$	0.002–1	$90^\circ$	0.1, 0, $-0.1$
Numerics-Cébron et al. (2019)	$\ge 10^{-5}$	$10^{-4}$–20	$18.5^\circ$–$161.5^\circ$	0
Numerics-Reddy et al. (2018), Komoda and Goto (2019)	$10^{-4}$	0.08–2	$90^\circ$	0–0.2
Dresdyn-Giesecke et al. (2018)	$\gtrsim 3\times 10^{-9}$	$< 0.1$	$45^\circ$–$90^\circ$	Cylinder

**Fig. 2:**
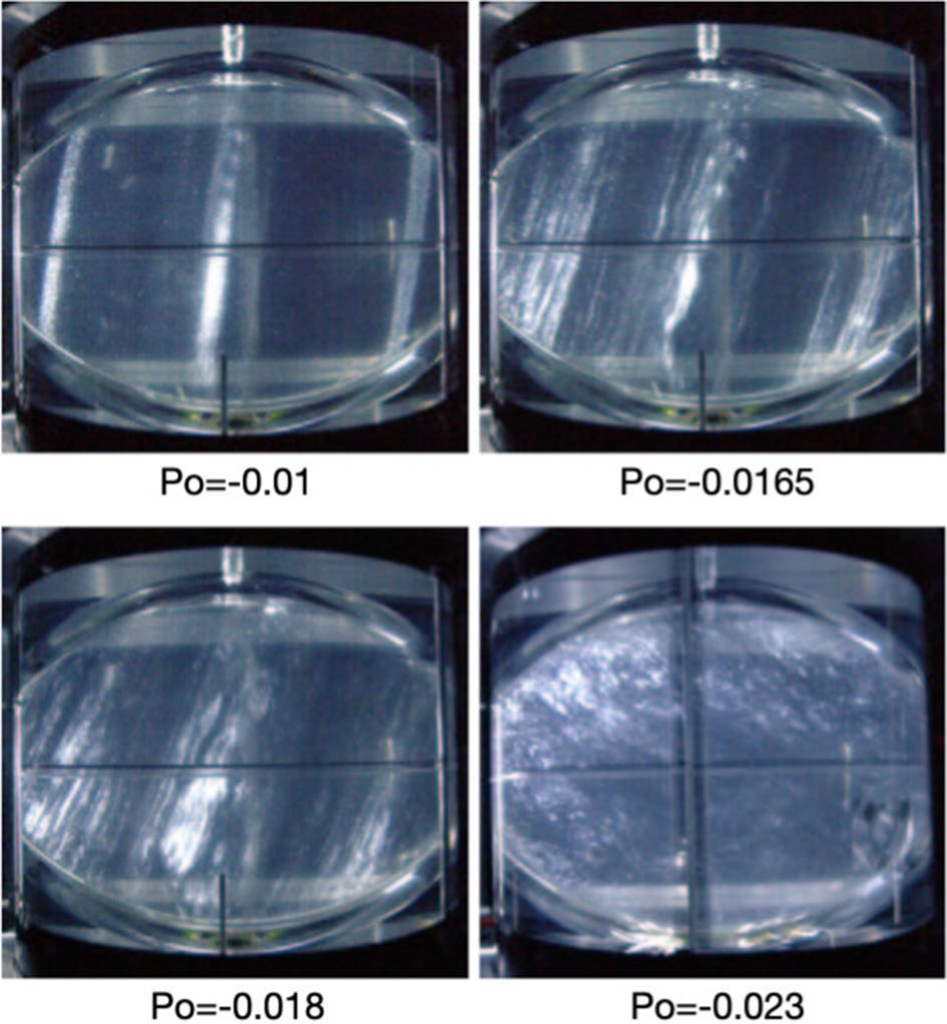
Visualizations in a meridional plane of the experiment by Noir et al. (2001) for increasing (in absolute value) precession forcing, illustrating the transition from the laminar base flow with a uniform vorticity flow along an inclined axis (top left), to the bulk filling turbulence (bottom right). Water is seeded with small reflective flakes called Kalliroscope that align preferentially in the flow due to their anisotropy. Lighted here by a meridional light sheet and observed from an angle of $90^\circ$, Kalliroscope emphasizes preferential domains of the flow, including zones of strong shear. The two steady, geostrophic bands symmetric with respect to the fluid rotation axis in the top left figure result from the non-linear interaction of the conical shear layers emitted at $30^\circ$ (oscillating flows within conical shear layers are not directly visible using Kalliroscope)

The pioneering precession experiments in spheroidal containers were performed by Willem Malkus in 1968 (Malkus 1968). He proposed that turbulent driven motions could power the geodynamo. He observed that laminar flow yields turbulence even at low precession rates (see also Fig. 2). In addition, he reported intense axisymmetric internal jets or shear layers, some of which were explained by Busse the same year (Busse 1968) as the result of nonlinear interactions in the boundary layer. These experimental results supported the possibility of turbulent motion driven by precession, opening the possibility for precession to power a dynamo in a liquid core. This pioneering work has been subject to controversy on the basis of erroneous energetic arguments (see the relevant discussion and correct argumentation in Kerswell 1996). Although we now know that Earth’s precession is too weak at present to drive the geodynamo, precession has been put forward as a plausible mechanism to power the past lunar dynamo (Dwyer et al. 2011).

This first discovery prompted other experiments in spheroids or spheres. In particular, Vanyo et al. in a series of well-designed experiments (Vanyo and Likins 1971; Vanyo 1973; Vanyo et al. 1995) illustrated a rich variety of flows occurring in a precessing spheroid. The extrapolation to geophysical objects attempted in some of these papers (Vanyo et al. 1995; Vanyo and Dunn 2000) is, however, erroneous. These experiments have later been interpreted in terms of laminar theoretical flow (Pais and Le Mouël 2001), and one particular pattern matches an important instability (see below) which was evidenced later (Lin et al. 2015). The various experiments by Vanyo et al. also showed that under certain circumstances energy dissipation in precessing cavities can be orders of magnitude larger than that predicted from a simple model of laminar viscous friction in the Ekman boundary layer, supporting the idea of dissipation enhanced by turbulence in precessing fluid cavities. Later, Goto et al. (2014) performed precession experiments in both a sphere and a spheroid at various Poincaré and Ekman numbers, with a fixed $90^\circ$ angle between $\varOmega _p$ and $\varOmega _s$. They highlight an optimal precession rate $Po=0.1$ to generate turbulence. In the range of parameters investigated in their study, the transition to turbulence in the spheroid happens at much larger *Po* than in the sphere. However, the resulting turbulence intensity and patterns are very similar in both the sphere and the spheroid and do not depend much on *Ek*. The reason turbulence may appear at larger *Po* in the spheroid is that, for a given *Po*, the weak viscous torque does not couple the liquid and the shell efficiently, leading to large differential rotation $d\omega$ in the sphere, which is prone to instabilities both in the boundary layer and in the bulk (see below). Conversely, the action of the pressure torque in the spheroid is more efficient at aligning the fluid and mantle rotation vectors, generating smaller differential rotation and thus more stable flows. The differential rotation between the Earth’s core and mantle is too small to reach the turbulent regimes reported by Vanyo and Goto, but recent numerical simulations and astronomical observations suggest that it is likely the case in the lunar core (Yoder 1981; Lin et al. 2015; Cébron et al. 2019).

Theoretical progress, experiments with improved measurements, and the increase in performance of numerical simulations in the last 10 years, shed light on the nature of the instabilities witnessed by Malkus, Vanyo, and Noir and helped to provide scaling laws for the onset of the different regimes. One can distinguish essentially three instability mechanisms: parametric instabilities in non-spherical cavities (Kerswell 1993), shear-driven bulk parametric instabilities (CSI) (Lin et al. 2015; Nobili et al. 2021), and boundary layer turbulence (Sous et al. 2013; Cébron et al. 2019).

The first mechanism results from the periodic shear and elliptical distortion of the circular streamlines of the solid body rotation in non-spherical containers, which resonantly couple two inertial waves. The onset of the most unstable mode was first derived by Kerswell (1993), who estimated that the critical value for the normalized differential rotation $d\omega$ between core and mantle is given by

4 $$\begin{aligned} d\omega _c\propto Ek^{1/2}\eta ^{-1}. \end{aligned}$$

It is thought to be the instability mechanism underlying the observations of Malkus (1968) and Goto et al. (2014) with large polar flattening $\eta = 0.1$. For real planets, the polar flattening is much smaller (see Table 1) and the critical values are therefore much larger. For example, for Earth, the differential rotation is estimated to be $d\omega \sim 10^{-7},$ while the critical value for the onset of the instability is $d\omega _c\sim 3 \times 10^{-5}$. The parametric instabilities are therefore unlikely to play any role on Earth today.

The shear-driven bulk parametric instabilities are due to oblique shear layers coupling with two free inertial modes. These conical shear layers are spawned from the so-called critical latitudes at $\pm 30^\circ$ where the flux from the Ekman boundary layer increases from $Ek^{1/2}$ to $Ek^{1/5}$ over a latitudinal extent proportional to $Ek^{1/5}$ (Stewartson and Roberts 1963). These local velocity and pressure perturbations propagate throughout the fluid interior as trains of inertial waves in the form of oscillating conical shear layers, forming an angle of $30^\circ$ with the rotation axis. Noir et al. (2001) confirmed experimentally and numerically the theoretical scaling of Stewartson and Roberts (1963) which allowed Lin et al. (2015) to derive the onset conditions using heuristic arguments: $d\omega _c\propto Ek^{3/10}$. This scaling law is in good agreement with the transition observed by Goto et al. (2014) and Horimoto et al. (2020) in precession experiments in a sphere. Scaled to Earth’s core, conical shear layers have flow velocities of the order of $10^{-6}$m/s over a width of 20 km, 100 times smaller than the velocity at the top of the core deduced from the secular variation of the geomagnetic field. They are also stable because the actual differential rotation $d\omega \sim 10^{-7}$ is two orders of magnitude smaller than the one required for instabilities. In contrast, for the lunar core, $d\omega \sim 10^{-3}$ and $10^{-5}<d\omega _c< 10^{-4}$ depending on the core size, suggesting that this mechanism could drive bulk instabilities (Lin et al. 2015).

The third and last mechanism, the shear boundary layer instability, has yet to be investigated experimentally in a precessing cavity. The theoretical onset condition is given by $d\omega _c \sim 50 Ek^{1/2}$ and, for emergence of a turbulent saturation, by $d\omega _c \sim 150 Ek^{1/2}$ for steady boundary layer [see (Cébron et al. 2019) and Sect. 5] and $d\omega _c \sim 500 Ek^{1/2}$ when oscillatory (Buffett 2021).

Comparing them with the threshold for the parametric instability (4), it seems difficult to disentangle these two sources of instability when the polar flattening is of the order $\eta \sim 1/150{-}1/50$, which is the case for the setups of Vanyo et al. (1995), Vanyo and Dunn (2000), and Noir et al. (2001) (see Table 1). The turbulence onset estimate for the Earth’s core yields $d\omega _c \sim 50 \times 10^{-7}$, a value closer, but still larger, than the present day estimate $d\omega \sim 10^{-7}$.

Spheroidal cavities exhibit hysteresis cycles of turbulence with respect to the precession rate *Po* near the resonance with the FCN. As first evidenced by Malkus (1968) and more recently by Nobili et al. (2021), it is closely related to the hysteresis of the differential rotation well known theoretically in spheroids (Cébron 2015). Starting from a laminar regime at low Po the differential rotation jumps to a much larger value (decoupled core–mantle) as the precession rate approaches the FCN frequency, leading to turbulent flows. Starting from the turbulent regime and decreasing the precession rate, the differential rotation can remain large even for $\varOmega _p<\varOmega _{FCN}$ and turbulence can be maintained. This has geophysical implications when a planet evolves from an early stage of large-amplitude orbital forcing to a more quiet epoch, which may have been the case for the lunar core (Cébron et al. 2019).

Extrapolation from laboratory settings to planetary cores requires assuming that experiments operate in the same asymptotic regime as planetary cores, which has yet to be proven. In fact, some recent studies (Le Reun et al. 2019; Lemasquerier et al. 2017) in the related case of libration (see Sect. 3) suggest that the dynamics observed in experiments at moderate Ekman numbers may be in a different regime than planetary cores. Furthermore, most experiments are performed in a range of Ekman numbers where onsets of all three types of instabilities described above are similar, so that several instabilities may be present at the same time and interact with each other (see, e.g., Nobili et al. 2021). One may thus wish to turn to numerical simulations of precession to obtain more definitive answers. While simulations start to become competitive with experiments in spherical geometry (see Table 1), the experimentally accessible range of parameters remains out of reach to numerics for spheroidal or ellipsoidal geometry.

These last comments call for a new generation of experiments operating at Ekman numbers $E\le 10^{-8}$. While it may look like a modest step, it means experiments of typically 3 m size rotating at 3 Hz. A rotating spherical Couette of such a size leads to much richer dynamics, as will be described in Sect. 4. Comparable size precessing experiments are even more challenging due to the extreme gyroscopic torque involved ($>10^6$ Nm). Such an ambitious experiment is currently under construction at the Helmholtz–Zentrum in Dresden, the so-called Dresdyn experiment (Giesecke et al. 2018), with the aim to investigate precession-driven dynamo processes (see targeted parameters in Table 1). Expected to operate in the coming years, it may bring forth new physical insights on the hydrodynamical regimes sketched above.

## Flows Driven by Libration

Longitudinal librations are periodic oscillations of a planet spin rate. We refer here to real, physical librations of a non-axisymmetric body due to gravitational interactions with its closest neighbors; they should not be mixed up with the so-called optical librations, which are just of observational origin (see, e.g., discussion in Noir et al. 2012). Numerous bodies of the Solar System undergo continuous, forced longitudinal librations including Mercury, the Earth’s Moon, the four Galilean satellites, etc. Libration can also be transiently excited or reinforced following a meteorite impact (Wieczorek and Le Feuvre 2009). Precisely measuring a planet’s forced libration provides constraints on its interior structure. For instance, a libration amplitude larger than predicted by models considering a fully solid planet is a clear signature for the existence of a liquid layer that decouples the interior from the shell. The libration amplitudes strongly suggest that at least part of Mercury’s core is still liquid (Margot et al. 2007) and that Europa has a liquid ocean underneath its thick ice shell (Van Hoolst et al. 2008). Most celestial mechanics models only account for the viscous torque that arises between the rotating fluid and the librating solid boundaries through laminar, or even turbulent, Ekman boundary layers (Yoder 1981). But beyond this, libration also excites bulk flows, similarly to other types of small harmonic forcing (precession, tides, etc., see, e.g., Le Bars et al. 2015). These flows have been suggested as possible sources for present dynamo action on Io (Kerswell and Malkus 1998) and for the past dynamo on the Moon (Le Bars et al. 2011), where the classical convective dynamo model fails to explain the observations. They also constitute a source of viscous dissipation and might significantly participate in the heat budget and orbital evolution of, for example, Enceladus (Wilson and Kerswell 2018; Rekier et al. 2019).

**Fig. 3:**
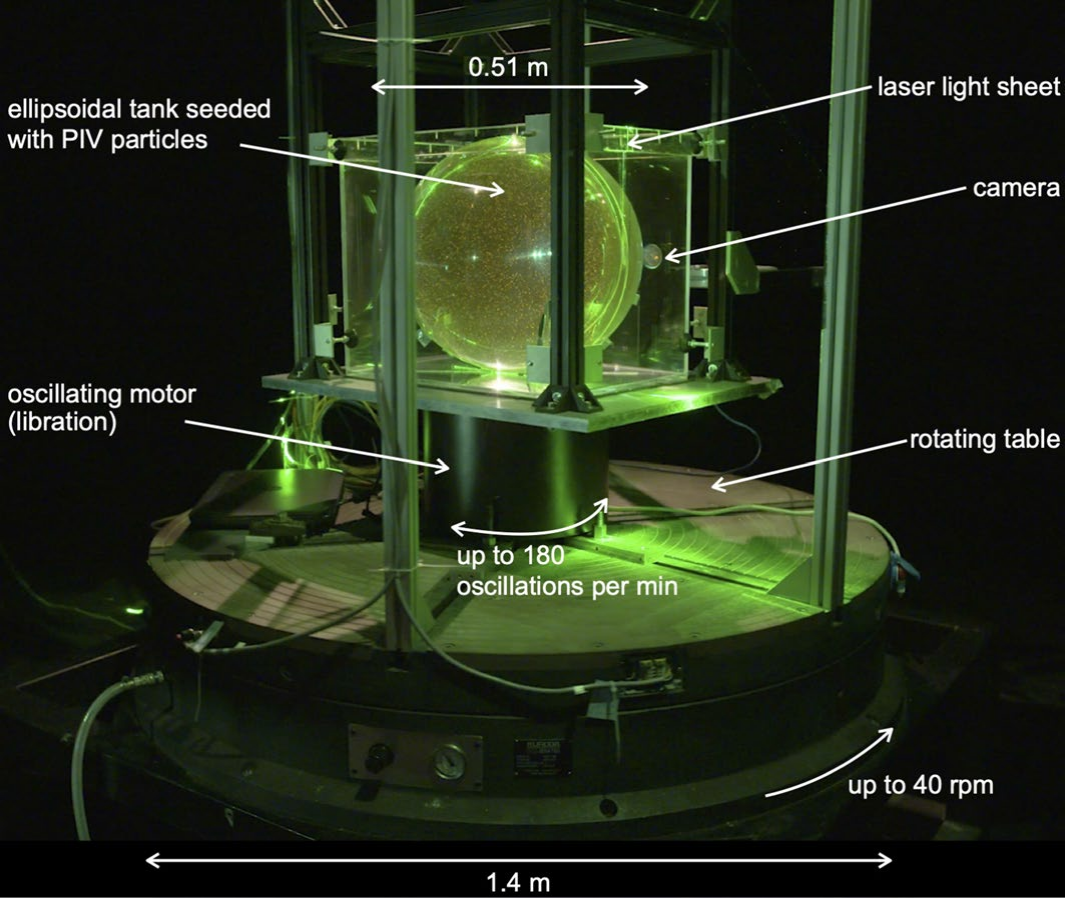
Experimental setup at IRPHE, Marseille (Le Reun et al. 2019), derived from the original design of Noir et al. (2009) and Noir et al. (2012). This large ellipsoidal installation allows exploring Ekman numbers down to $Ek=3.7\times 10^{-6}$ with turbulent flows down to an excitation Rossby number $Ro=3.4\times 10^{-2}$, defined here as the product of the libration amplitude times the ellipticity in the equatorial plane. Hence, this setup allows characterizing the transition between the 2D geostrophic turbulence at relatively large *Ro* and the asymptotic, 3D wave turbulence regime at small *Ro*. See also online movie at https://www.youtube.com/watch?v=Drq2qxX0U90

Laboratory experiments have contributed to deciphering and characterizing the variety of libration-driven flows. In such experiments, a hollow container, filled most of time with water, is set in rapid rotation by a first motor; librations are generated either by modulating the spin rate of this first motor through time, or more efficiently by a second onboard motor that oscillates the outer and/or inner boundary of the container (see, e.g., Fig. 3). Studies are then performed by systematically changing the global rotation rate quantified non-dimensionally by the Ekman number, and/or the libration amplitude quantified by the Rossby number, and/or the ratio between the libration frequency and the spin rate. Axisymmetric as well as non-axisymmetric containers have been considered. Metrology includes local pressure measurements (Aldridge and Toomre 1969), Kalliroscope visualizations (Noir et al. 2009; Koch et al. 2013; Lemasquerier et al. 2017), as well as non-intrusive velocimetry by LDA (Noir et al. 2012) and PIV (Sauret et al. 2010; Koch et al. 2013; Grannan et al. 2014; Hoff et al. 2016; Kozlov and Subbotin 2017; Lemasquerier et al. 2017; Le Reun et al. 2019). Main parameters of these various experimental setups are listed in Table 2.

**Table 2:** Main characteristics and parameters of various setups designed for experimental studies of libration

Reference	Geometry	Ekman number	Libration amplitude	Libration frequency
Noir et al. (2012), Grannan et al. (2014), Lemasquerier et al. (2017)	Sphere, (half) prolate spheroid ($\beta =0.06, 0.34$), prolate spheroid shell ($\beta =0.34$)	$2 \times 10^{-5}$	0–2.5	0.5–2
Le Reun et al. (2019)	Triaxial ellipsoid ($\beta =0.34$)	$3.7 \times 10^{-6}$	0.075–0.331	4
Noir et al. (2009)	Sphere, spherical shell	$10^{-5}$	0.03–5	0–1
Aldridge and Toomre (1969)	Sphere	$7.5 \times 10^{-6}$	0.07–0.28	0.5–2
Koch et al. (2013)	Spherical shell	$2.5 \times 10^{-5}$	0.1–1	0.2–2
Sauret et al. (2010)	Sphere	$10^{-5}$	0.02–0.15	0.04–0.1
Hoff et al. (2016)	Spherical shell	$1.6 \times 10^{-5}$	0–2	0–1
Kozlov and Subbotin (2017)	Spherical shell with free inner core	$6 \times 10^{-5}$	0–0.5	0–2.5
Enceladus ocean	Triaxial ellipsoid ($\beta =2\times 10^{-2}$)	$3.4 \times 10^{-14}$	$2.1 \times 10^{-4}$	2

Here, libration frequency is non-dimensionalized by the mean spin rate, and libration amplitude is equal to the angular half-amplitude times this dimensionless libration frequency. $\beta$ is the ellipticity in the equatorial plane. Values for Enceladus subsurface ocean are also provided as an example; more data for planets and moons can be found in Cébron et al. (2012) and Lemasquerier et al. (2017)

**Fig. 4:**
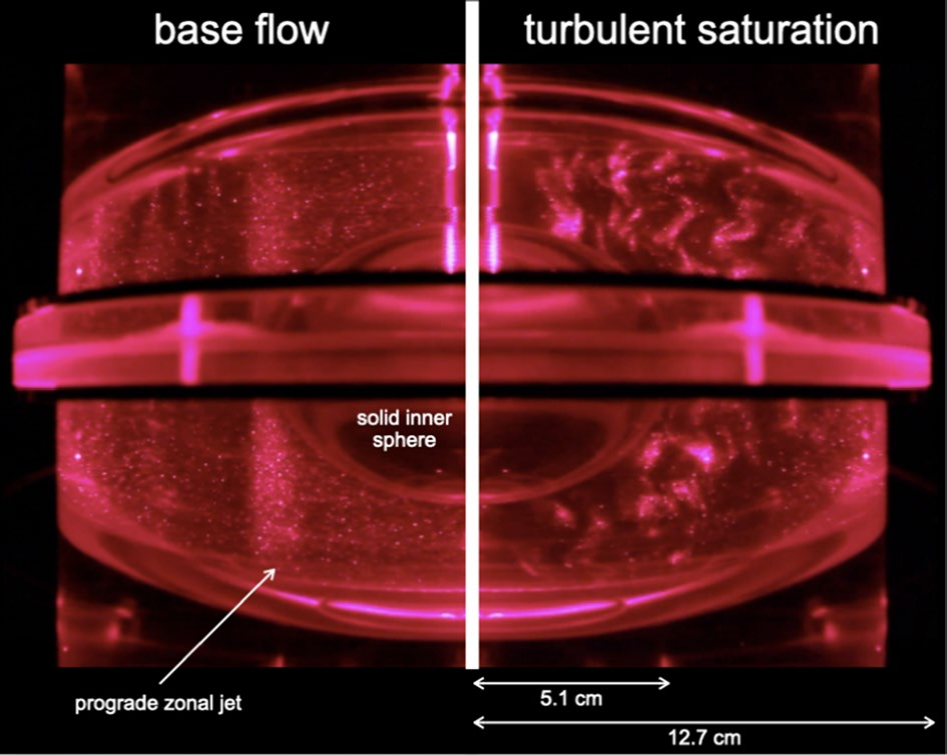
Kalliroscope visualizations in a meridional plane of an experimental run in the UCLA ellipsoidal shell setup (Lemasquerier et al. 2017), with a rotation rate of 35 rpm, a libration angle of $7.5^\circ,$ and a libration frequency of four times the rotation rate: These correspond to an Ekman number $Ek=2.6\times 10^{-5}$ and an excitation Rossby number $Ro=0.18$ (equal to the product of the libration amplitude times the ellipticity in the equatorial plane). The left picture shows the initial base flow, with the noticeable Stewartson layer jet aligned with the rotation axis and tangent to the solid inner sphere. The right picture shows the turbulent saturation that settles a few tens of seconds later, with noticeable small-scale, 3D wavy patterns. See also online movie at https://www.youtube.com/watch?v=WGe-vLsm9Ho

Focusing here on the spherical and ellipsoidal geometries relevant for planets, the first experimental study dates back to 1969, when Aldridge and Toomre (1969) used libration forcing to systematically assess the direct resonance of axisymmetric, inertial modes in a rotating sphere. Very schematically, librating an axisymmetric container filled with fluid in solid body rotation a priori only affects its Ekman layer, while its interior remains in solid body rotation. However, for any given libration frequency within the domain of existence of inertial modes (i.e., between 0 and 2 in dimensionless form, see Sect. 1), the Ekman layer exhibits a singularity at a critical angle, and this eruption excites an inertial mode with the same frequency within the bulk (Le Bars et al. 2015). This mechanism somehow generalizes the excitation of conical shear layers at $30^\circ$ in precessing flows (see Sect. 2). The efficiency of this resonance mechanism has been challenged in the limit of small Ekman and Rossby numbers (Zhang et al. 2013); it was, however, clearly observed in Aldridge and Toomre (1969). Aldridge’s work was then extended to spherical shells by Koch et al. (2013), Hoff et al. (2016), and Lemasquerier et al. (2017), who reported more intense inertial mode resonances due to energy focusing toward wave attractors. Those studies also systematically reported the emergence of a prograde zonal jet within the Stewartson layer along the tangent cylinder (see Fig. 4 left). As for the well-known Stokes drift associated with surface water waves, the formation of a steady, geostrophic flow from an oscillatory forcing is due to nonlinear interactions, in those cases of the oscillating flow within the Ekman layer due to the libration of the inner core. Even more interesting for dynamo and dissipation applications, the Stewartson layer jet becomes unstable at sufficiently large forcing amplitude, generating bulk filling turbulence from centrifugal and shear instabilities as well as through nonlinear inertial wave interactions (Hoff et al. 2016).

Beyond this Stewartson layer jet, the emergence of steady zonal flows in librating systems has been studied in great detail. Even away from any resonance, a global flow takes shape systematically over the whole bulk because of nonlinear interactions of the libration base flow within the Ekman layer, as first shown theoretically by Busse (2010) and validated experimentally by Sauret et al. (2010) in the spherical geometry. Zonal flows also exist in more complex geometries like ellipsoids (Noir et al. 2012). With a free, floating, solid inner core and a librating outer shell, zonal flows induce inner core differential rotation by viscous coupling (Kozlov and Subbotin 2017). Additionally, strong, localized zonal jets might superimpose on this global zonal flow when inertial waves are excited, due to their nonlinear self-interactions within the Ekman layer around their reflection points (Calkins et al. 2010), as already described for precession in Sect. 2 and Fig. 2. Torques associated with those zonal flows and their consequences for the celestial motions of the planets remain to be investigated.

The librating flow in the Ekman layers includes successive spin-up and spin-down phases through time: Hence, no matter whether you consider the inner or outer boundary, it is always at some point prone to centrifugal instability. This mechanism has been studied by Noir et al. (2009) who showed, for increasing Rossby number, the destabilization of the laminar outer Ekman layer in a librating sphere, first by the emergence of longitudinal rolls, then by the settling of boundary turbulence. Numerous planets fall in one of those two regimes, including our Moon. The relevance of those boundary flows for planets where Ekman layers are very thin might be questioned. The interest is actually twofold: First, destabilization of the otherwise laminar Ekman layer might explain anomalous strong dissipation and associated faster than expected orbital evolution. This might for instance account for the rapid Moon recession measured presently by Lunar Laser Ranging (Williams et al. 2001). Second, turbulent motions in the boundary layer might excite inertial waves in the bulk that propagate and invade the whole interior, as shown in axisymmetric numerical simulations by Sauret et al. (2013): First-order consequences like dynamo (Moffatt 1970) and increased dissipation associated with bulk wave turbulence (Le Reun et al. 2019) might be expected from these extended flows. This could be the focus of future studies.

Intense wave dynamics in the bulk of planetary cores has been studied in the more readily applicable context of a librating ellipsoidal container: There, libration can excite a so-called parametric instability, similar to the first instability mechanism for precession described in Sect. 2. The basic mechanism for instability is a resonance between two inertial modes of the system and the libration base flow in the bulk induced by topographic coupling. This resonance requires that the difference between the frequencies of the two inertial modes equals the libration frequency. Acknowledging that inertial modes have frequencies between plus and minus twice the spin rate $\varOmega$ (see Sect. 1), resonances are possible for libration frequencies up to $4\varOmega$; selection of effectively emerging resonances then depends on the combined effects of the specific shape of the container and of the amplitude of viscous dissipation quantified by the Ekman number. The main interest for planetary applications follows from the underlying mechanism: The generated flow is due to an instability, meaning that the associated energy grows exponentially in time until it saturates at a given value; and the generated flow is related to a resonance, meaning that the saturation amplitude can be much larger than the excitation. Small amplitude, libration forcing (*Ro*, defined here as the product of the libration amplitude times the equatorial ellipticity, is typically of order $10^{-4}$ or less in planets and moons, see Table 2 and Noir et al. 2012; Cébron et al. 2012; Lemasquerier et al. 2017) allows the flow to draw energy from the huge reservoir available from the rotation of the planet. This energy can then be used to sustain the inertial modes which are the building blocks of this process. This so-called libration-driven instability was first observed indirectly in experiments, through LDA measurements of significantly increased zonal flows for selected libration frequency ranges in a librating ellipsoid (Noir et al. 2012). Using the same setup, Grannan et al. (2014), followed by Lemasquerier et al. (2017) in the ellipsoidal shell, then performed systematic exploration of the parameter space and quantified various possible resonances in the expected range of libration frequency, Rossby number, and Ekman number. After the documented exponential growth, the flow at saturation was described as a superposition of columnar zonal flows and tri-dimensional wavy patterns due to inertial waves (see, e.g., Fig. 4 right), with either cycles of growth and collapse or saturation around a mean chaotic state.

In fact, the flow layout at saturation of this instability is a complex, open question that echoes the broader, long-standing issue of rotating turbulence (Godeferd and Moisy 2015). There are (at least) two different models for turbulence in rotating fluids. In the first model called quasi-geostrophic turbulence, energy progressively concentrates in quasi-2D flows aligned with the rotation axis, like the zonal jet described above. The quasi-2D dynamics then leads to the formation of large columnar structures by inverse cascade (Godeferd and Moisy 2015). This quasi-geostrophic turbulence is for instance characteristic of rapidly rotating convective regimes relevant for planetary convective cores (Guervilly et al. 2019). In the second model called inertial wave turbulence, energy transfers are due to successive triadic interactions where, somehow similarly to the process of parametric resonance already described for the onset of both precession and libration instabilities, each given inertial mode excites nonlinearly two additional, resonant ones: Motions then remain fully tri-dimensional, made of a superposition of weakly nonlinearly interacting inertial modes. Most experiments of rotating turbulence where energy is injected by grids, jets, flaps, etc., exhibit geostrophic saturation (Godeferd and Moisy 2015). But in the special case of libration, where energy is injected predominantly in inertial modes, it was recently demonstrated experimentally using a dedicated, large installation (see Fig. 3) that the saturation depends on the forcing amplitude: Classical quasi-geostrophic turbulence settles at relatively large Rossby numbers, while wave turbulence settles in the planetary relevant limit of small Rossby numbers (Le Reun et al. 2019). This conclusion completely changes our view of non-convecting cores and implies that our classical estimates for dissipation and dynamo capacity were mostly based on quasi-geostrophic, convective-like flows (Le Bars et al. 2011; Wu and Roberts 2013; Reddy et al. 2018), should be re-examined.

Clearly, the study of libration-driven flows and their consequences still has a future ahead. In particular, the question remains open to demonstrate whether or not inertial wave turbulence is capable of generating a large-scale planetary magnetic field, following the promising theoretical model of Moffatt (1970). Also, combining libration forcing with buoyancy effects, including either convectively unstable or stably stratified configurations, is both experimentally challenging and of great relevance for planetary applications.

## Spherical Couette Flows

**Fig. 5:**
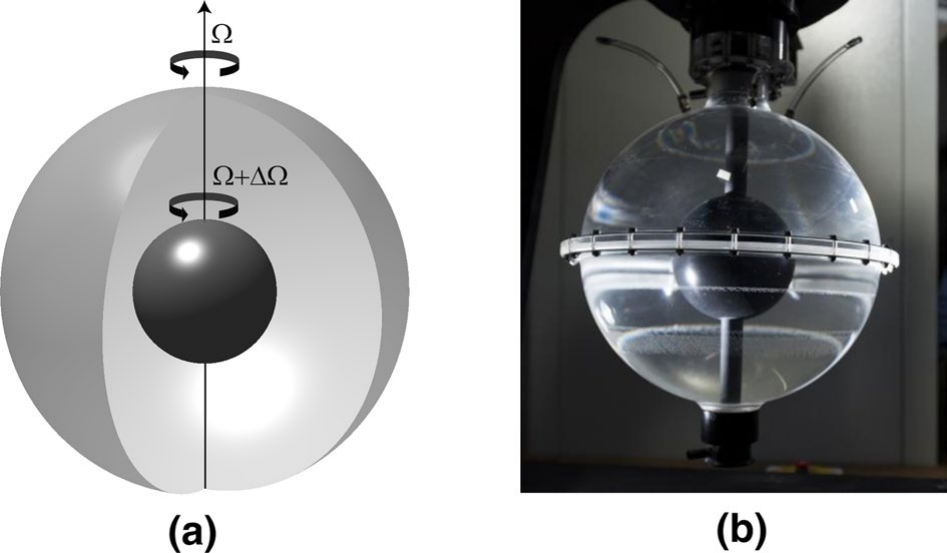
The spherical Couette system—two differentially rotating spherical shells with fluid between them. **a** a schematic of the system, **b** the Cottbus spherical Couette setup (*Source*: Michael Hoff)

The spherical Couette system consists of two concentric differentially rotating spheres, the space in between filled with a fluid (Fig. 5a). This is the spherical equivalent of the well-studied Taylor–Couette system with two concentric cylinders. Interiors of compact astrophysical objects such as planets and stars have geometries similar to the spherical Couette configuration. The Sun’s convective zone, the Earth’s core, and the interior of the giant planets are prime examples. It is probably the simplest laboratory model to study the influence of both rotation and shear on fluid dynamics, which are two ubiquitous phenomenons found in geophysical and astrophysical objects. For instance, in planets like Jupiter or Saturn the differential rotation manifests as alternating zonal winds in the atmosphere. The Sun, similarly, exhibits a convective zone where the equatorial regions rotate faster than the polar regions. It is important to note, however, that the aim of the various spherical Couette experiments in existence is to understand the basic and fundamental questions posed by rotating flow phenomena, rather than serving as accurate replicas of the above-mentioned astrophysical objects. Furthermore, the spherical Couette system was also considered as a viable candidate for experimental dynamo action in a geophysically relevant geometry, in contrast to thermal convection which cannot produce vigorous enough flows in the laboratory.

In the geophysical context where the influence of rotation is crucial, dynamicists are interested in the case where the outer container is rapidly rotating. As with many rotating systems, spherical Couette flow is then studied from a frame of reference rotating with the outer container. The fluid in the spherical Couette system is driven viscously via the differential rotation due to the inner sphere, which may rotate faster, slower, or in opposite direction to the rotation of the outer container. Instabilities arise depending on the amount and direction of the differential rotation. There are three basic parameters—the aspect (radius) ratio ($r_i/R$) determining the size of the inner sphere relative to the outer one, the outer boundary rotation ($\varOmega$) expressed non-dimensionally by the Ekman number (see definition 1), and the differential rotation between the two spheres. The latter is usually expressed as a ratio to the outer boundary rotation rate ($\varDelta \varOmega /\varOmega$, equivalent here to a Rossby number, see definition 2), with positive differential rotation referring to the case where the inner sphere rotates faster while negative referring to the case where the inner sphere rotates slower than or in the opposite direction to the outer sphere.

As a classical fluid mechanical system, the spherical Couette system has been studied since the 1950s using analytical methods (Proudman 1956; Bratukhin 1961; Stewartson 1966; Starchenko 1997; Kleeorin et al. 1997), experiments (Sorokin et al. 1966; Munson and Menguturk 1975; Belyaev et al. 1978; Yavorskaya and Belyaev 1986; Egbers and Rath 1995; Hollerbach et al. 2004) as well as numerical computations (Hollerbach et al. 2004; Munson and Joseph 1971a, b; Hollerbach 1994, 2000; Hollerbach and Skinner 2001; Hollerbach 2001, 2003; Hollerbach et al. 2006; Hollerbach 2009). In more recent years, a new generation of hydrodynamic, dynamo, and magnetohydrodynamic (MHD) experiments are using this setup, aimed at studying flows, instabilities, and turbulence in the outer core of the Earth (Sisan et al. 2004; Kelley et al. 2007, 2010; Zimmerman et al. 2011; Triana 2011; Rieutord et al. 2012; Zimmerman et al. 2014; Nataf et al. 2006; Schmitt et al. 2008; Brito et al. 2011; Schmitt et al. 2013; Tigrine et al. 2019; Kasprzyk et al. 2017; Garcia et al. 2019, 2020, 2020; Koch et al. 2013; Hoff et al. 2016; Hoff and Harlander 2019), a list of which is provided in Table 3. Their focus is on “wide-gap” aspect ratios close to that of the Earth’s outer core ($r_i/R = 0.35$).

These have been complemented as well as supplemented by advanced numerical studies (Rieutord et al. 2012; Tigrine et al. 2019; Matsui et al. 2011; Wicht 2014; Barik et al. 2018; Kaplan et al. 2018). Most experimental studies take place at low Ekman numbers, typically $Ek < 10^{-4}$ with the 3-meter experiment (discussed below) reaching values of $Ek\sim 10^{-8}$. The large range of time and length scales excited by turbulence at these parameters makes direct numerical simulations (DNS) of unstable flows difficult. Thus, studies at high Ekman numbers are typically done using numerical methods, while studies at very low Ekman numbers are only possible using experiments. In between, there is a narrow range of $10^{-5}< Ek < 10^{-4}$, where both experiments and DNS overlap (e.g., Barik et al. 2018). Thus, the spherical Couette system provides a framework where numerical and experimental studies can come together to study fundamental flows and instabilities applicable to interiors of spherical compact astrophysical objects.

Here, we will discuss recent insights into the system obtained from the aforementioned experimental and numerical studies. We begin our survey, which is not meant to be exhaustive, by presenting results dealing with purely hydrodynamical phenomena.

**Table 3:** List of the spherical Couette experiments discussed in the text

Experiment	Working fluid	Diameter (2*R*)	*Ek* order	Radius ratio ($r_i/R$)
30 cm	Liquid sodium	30 cm	$10^{-7}$*	0.35
60 cm	Liquid sodium	60 cm	$10^{-7}$	0.35
3 meter	Water/liquid sodium	3 m	$10^{-8}$	0.35
DTS	Liquid sodium	42 cm	$10^{-7}$	0.35
HEDGEHOG	GaInSn alloy	18 cm	$10^{-4}$*	0.33, 0.5
Cottbus experiment	Silicone oil	24 cm	$10^{-5}$	0.33

*Means that the experiments were run with outer sphere stationary. *Ek* for these cases is based on inner boundary rotation rate and radius and thus represents the inverse of the Reynolds number at inner boundary [see definitions (1,3)]

### Hydrodynamic Experiments and Instabilities

The general class of hydrodynamic instabilities in the spherical Couette system is first determined by the radius ratio. When the gap between the two spheres is narrow ($r_i/R \ge \approx 0.7$), instabilities in the spherical Couette flow are similar in form to Taylor vortices near the equator, as found in the classical cylindrical Taylor–Couette flow (Egbers and Rath 1995; Soward and Bassom 2016). They are due to a centrifugal instability characterized by the Rayleigh criterion (Rayleigh 1917). However, for the more geophysically relevant wide gap, which we would discuss here, the primary linear instabilities are fundamentally different than Taylor vortices, instead forming drifting waves due to a shear instability (Egbers and Rath 1995; Hollerbach et al. 2004; Hollerbach 2003; Wicht 2014). In addition, as described in introduction (Sect. 1), the overall rotation of the system (except for $\varOmega =0$) greatly modifies the flow, generally at the larger length scales. Typical modifications for rapidly rotating flows include large-scale waves, zonal flows, and rotationally modified boundary layers (Ekman layers). We now take a brief survey of different experiments and briefly discuss their results.

**Fig. 6:**
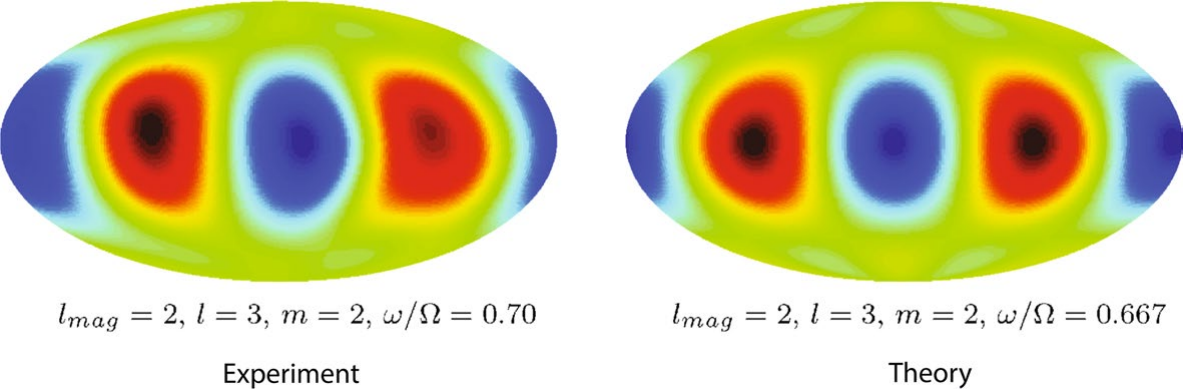
Inertial mode observed in the induced magnetic field in the 60-cm spherical Couette device. Here, the magnetic field is represented as an equal area projection at the outer sphere where blue is outward pointing magnetic field and red inward. The left image shows the observed magnetic field in a state with a dominant $l=3$ inertial mode; the right image shows the computed magnetic field that would be induced by a whole-sphere inertial mode of the same *l*, *m*. The frequencies of the experimentally observed and analytically computed modes are comparable. Adapted from Kelley et al. (2007)

We first discuss the University of Maryland’s 60-cm-diameter liquid sodium experiment (Kelley et al. 2007). Although the working fluid in this device was liquid sodium, which is an electrical conductor, and magnetic fields were used for diagnostics, the instabilities that appeared were purely hydrodynamic in origin: Both imposed and induced magnetic fields were indeed too weak to influence the flow. With a 60-cm outer sphere diameter and aspect ratio of $r_i/R=0.35$, this experiment could attain $Ek \sim 10^{-7}$. Two external Helmholtz coils provided an axial magnetic field parallel to the rotation axis. The flow of liquid sodium led to induced magnetic fields which were measured using arrays of Hall probes. The number and location of Hall probes allowed detecting flow structures corresponding to spherical harmonics up to degree $l=4$. For ${{\varDelta }{\varOmega }/{\varOmega }}< 0$, the magnetic Hall probes revealed oscillatory flow patterns typical of global inertial modes (see Sect. 1). The experimentally observed states were observed to be similar to the analytical inertial eigenmodes of a *full* sphere in uniform rotation (Fig. 6) (Zhang et al. 2001).

The same phenomena were observed in the 3-meter experiment also at the University of Maryland. Before being used for MHD and dynamo experiments, the 3-meter experiment used water as the working fluid. Essentially a scaled-up version of the 60-cm device, the 3-meter device is to date the largest spherical Couette facility in the world. For the water experiments, the flow diagnostics consisted of ultrasound Doppler velocimetry, torque measurements, wall shear stress hot film, and pressure measurements. As shown, for example, in Rieutord et al. (2012), inertial modes appeared in the 3-meter experiment at the same differential rotation parameters as in the 60-cm device. A few additional modes were observed which allowed a better characterization of the full set. The flow exhibits a diversity of different rotating turbulent states in addition to the inertial mode states, determined by differential rotation (Rossby number) alone.

**Fig. 7:**
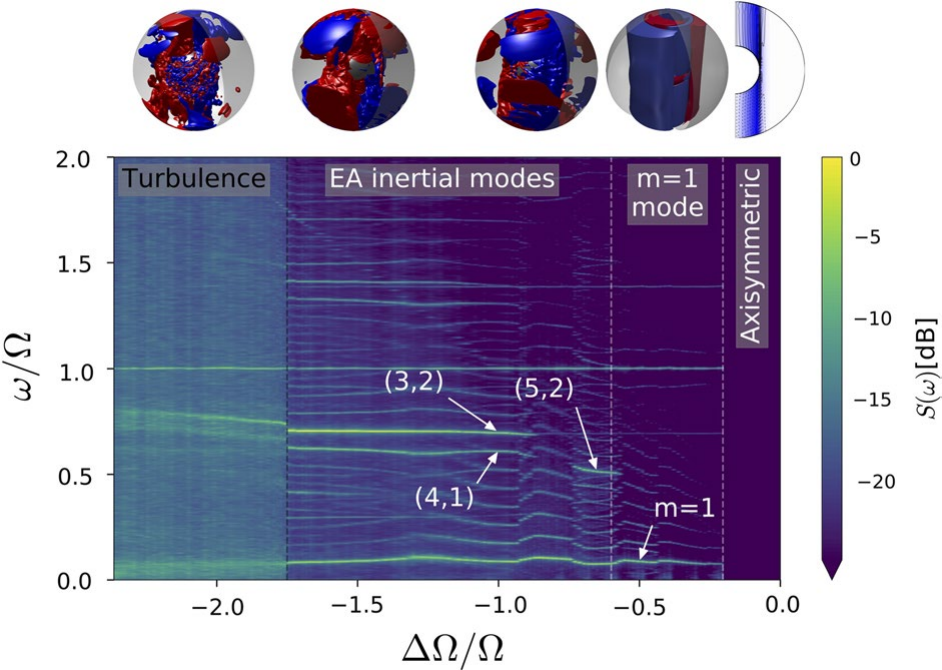
Spectrogram from PIV measurements performed on the Cottbus spherical Couette experiment (Hoff et al. 2016). The Ekman number is $Ek=1.52\times 10^{-5}$. EA means equatorial antisymmetric. Broadband background turbulence appears here when ${{\varDelta }{\varOmega }/{\varOmega }}\le -1.75$. The figures on the top show flow renderings from simulations (Barik et al. 2018)

The Cottbus spherical Couette experiment (Koch et al. 2013), with a 24-cm diameter outer sphere and $r_i/R = 1/3$, provided important insight into the behavior of the system at $Ek\sim 10^{-5}$, higher than in the Maryland experiments (Hoff et al. 2016; Hoff and Harlander 2019). The device employs a transparent silicone oil seeded with spherical hollow glass particles as tracers for particle image velocimetry measurements. For small absolute values of the Rossby number, the flow is axisymmetric and resembles the inviscid analytical solution by Proudman (1956) (the situation is different for high Ekman numbers (Bratukhin 1961; Wicht 2014) but that is beyond the scope of the present text). Outside the tangent cylinder (TC), the fluid stays in solid body rotation with the outer boundary, while inside the TC, the fluid rotates at half the differential rotation rate ($\varDelta \varOmega /2$). This leads to the formation of a nested Stewartson shear layer (Stewartson 1966) where instabilities at smaller Ekman numbers take place. When the counter-rotation in increased so that the negative Rossby number decreases below a critical value, this experiment found a primary instability with an azimuthal wave number $m=1$. Further increasing the magnitude of differential rotation increased the flow transitions to secondary instabilities and ultimately toward turbulence (Fig. 7). This experiment also found an onset of inertial modes, similar to the ones observed in the Maryland experiments. The phenomena at ${{\varDelta }{\varOmega }/{\varOmega }} < 0$ were reproduced by a detailed numerical study (Barik et al. 2018), which provided more insight into the excitation of inertial modes but also led to new questions. A wide ranging study of the different instability regimes as revealed by numerical experiments can be found in Wicht (2014).

It turns out that for moderately high outer boundary rotation ($3\times 10^{-5}< E < 10^{-3}$), the nature of the primary instability depends on the sign of the differential rotation, with high wavenumber instabilities occurring for positive differential rotation (${{\varDelta }{\varOmega }/{\varOmega }}>0$), while a wavenumber $m=1$ instability occurring for negative values (${{\varDelta }{\varOmega }/{\varOmega }}<0$). The reason for this dichotomy remains an open question (Hollerbach 2003; Hoff and Harlander 2019). The $m=1$ mode seems rather special. At lower Ekman numbers, it onsets as a secondary instability, shortly after the primary instability (Wicht 2014; Barik et al. 2018). The mode has also been observed at the extremely low Ekman numbers of the 3-meter experiment (Rieutord et al. 2012) and only for ${{\varDelta }{\varOmega }/{\varOmega }} < 0$. More negative differential rotation beyond the onset of the $m=1$ mode leads to the onset of equatorially antisymmetric inertial modes which lead to a spontaneous symmetry breaking of the flow. The origin of these modes leads to open questions about their onset for only negative differential rotation and the breaking of symmetry over a wide range of Ekman numbers from $10^{-4}$ (Hoff et al. 2016; Wicht 2014; Barik et al. 2018) to $10^{-8}$ (Kelley et al. 2007, 2010; Rieutord et al. 2012; Matsui et al. 2011), against an otherwise strong geostrophic constraint which, as explained in the introduction (Sect. 1), imposes quasi-invariance of the flow along the axis of rotation for low-frequency forcing. Further open questions include the selection of modes that onset and the accompanying triadic resonances (Hoff et al. 2016; Barik et al. 2018) that follow specific rules with respect to wavenumbers and azimuthal drift frequencies, as already discussed above for precession (Sect. 2) and libration (Sect. 3) forcings. A further increase in driving toward even more negative differential rotation values leads to inertial modes without triads and featureless turbulence which have their own unique properties.

As briefly mentioned above, ${{\varDelta }{\varOmega }/{\varOmega }}<0$ phenomena are very different compared to the ${{\varDelta }{\varOmega }/{\varOmega }}>0$ case. For instance, the 3-meter water experiments revealed a bi-stable turbulent state where the system switched over long periods of time between a high-torque state and a low-torque state (Zimmerman et al. 2011) as measured by a torque sensor on the inner sphere shaft. These transitions occurred over the range $1.7<{{\varDelta }{\varOmega }/{\varOmega }}<2.5$. The relative time spent in each of the low- and high-torque states varied smoothly across that ${{\varDelta }{\varOmega }/{\varOmega }}$ range. The switching between these two states, for a fixed ${{\varDelta }{\varOmega }/{\varOmega }}$, is a remarkable example of how planetary (rotating) flows may have multiple apparently statistically steady states. The switching in this case appears probabilistic, but with a specific probability of each state at a given ${{\varDelta }{\varOmega }/{\varOmega }}$. The transitions resemble outward outbursts of angular momentum. In the low-torque state, the fluid inside the tangent cylinder spins faster than the fluid outside, as if “detached” from it. During the sudden transition to the high-torque state, the velocity gradient across the TC is reduced. Sudden angular momentum outbursts, conceptually similar to the one described, occur also in astrophysical objects, e.g., the so-called *Be-phenomenon* where an almost critically spinning, type Be star ejects material that suddenly acquired too much angular momentum (Neiner and Mathis 2013). These outbursts might have a common origin with the ones observed in the 3-meter experiment. To date, both are still awaiting a satisfactory explanation.

### Hydromagnetic Experiments

**Fig. 8:**
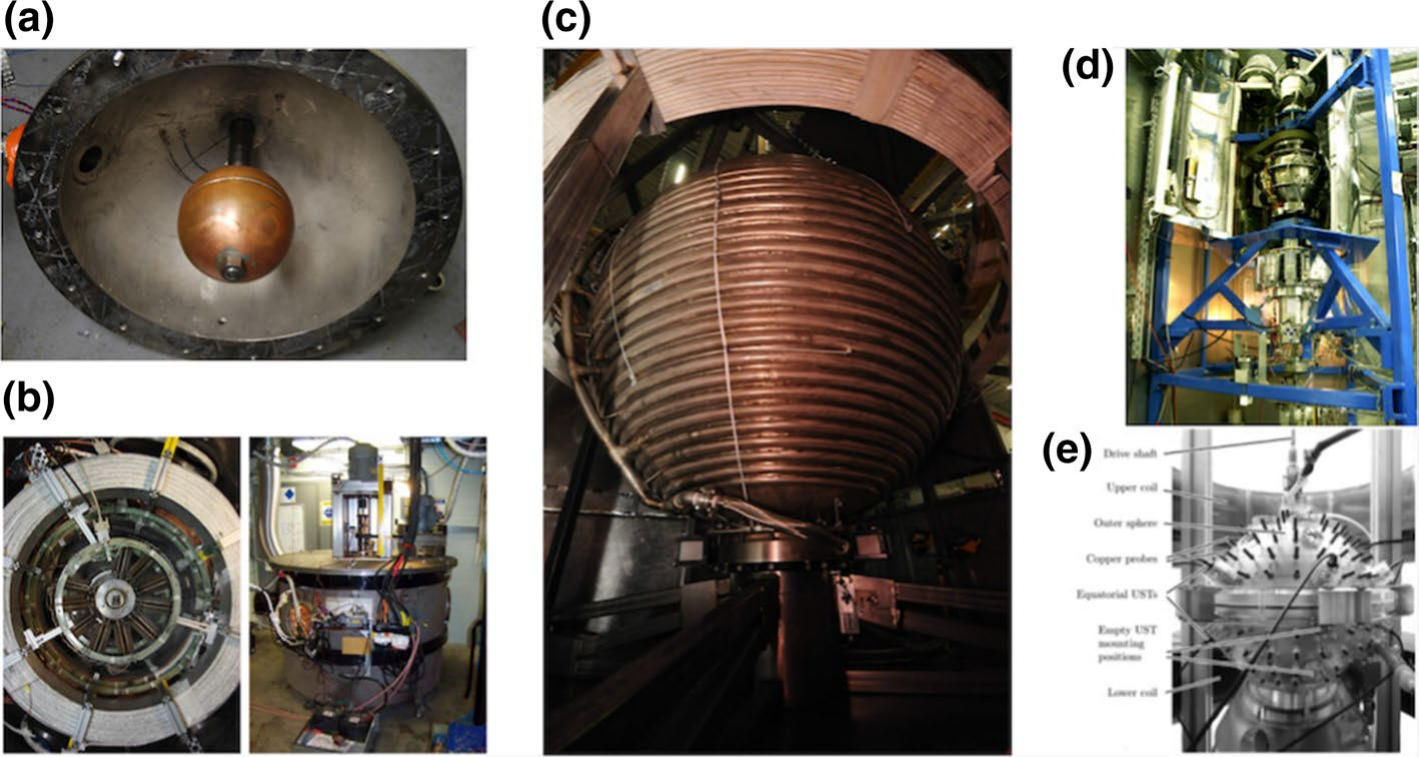
The hydromagnetic spherical Couette experiments: **a**–**c** the three Maryland experiments—30 cm, 60 cm, and 3 meter, respectively (*Sources*: S.A. Triana, Kelley 2009 and D. Lathrop). **d** DTS experiment (picture courtesy: H.-C. Nataf) and **e** HEDGEHOG experiment (figure from Kasprzyk et al. 2017). See also online movies of the 3-meter setup at https://youtu.be/bm_iqzmR2cE and https://youtu.be/rAYW9n8i-C4

The application of a background magnetic field introduces further complications in the spherical Couette flow of conducting fluids. The typical magnetic field configurations used in experiments are an axisymmetric axial field (Kelley et al. 2007; Garcia et al. 2019) or an axisymmetric dipolar field (Schmitt et al. 2008). As mentioned before, weak magnetic fields can be used for flow diagnostics, e.g., Kelley et al. (2007, 2010). When the hydrodynamic flow is axisymmetric, as the strength of the magnetic field increases, the Stewartson free shear layer gradually transitions to a magnetic free shear layer called a “Shercliff” layer (Hollerbach 1994; Kleeorin et al. 1997; Wei and Hollerbach 2008) and Ferraro’s isorotation law (Ferraro 1937) gradually replaces the Taylor–Proudman constraint. The exact form of the axisymmetric flow and Shercliff layer now additionally depends on the magnetic field topology and boundary conditions (conducting/insulating) on the two boundaries, and Lorentz forces may give rise to strong jets (Starchenko 1997; Dormy et al. 1998; Hollerbach 2000, 2001; Soward and Dormy 2010). Two new dimensionless numbers now become important. The first is the Hartmann number, $\mathrm {Ha}=B_0 r_i\sqrt{\sigma /\rho \nu }$ which describes the non-dimensional strength of the applied magnetic field $B_0$, where $\sigma$ and $\rho$ are, respectively, the fluid electrical conductivity and density. The boundary layers gradually transform from Ekman layers into Ekman–Hartmann layers (Hollerbach 1994) whose thickness is eventually determined by $\mathrm {Ha}$. The second is the magnetic Prandtl number, $\mathrm {Pm}=\mu _0\nu \sigma$, non-dimensionally describing the fluid viscosity relative to the electrical resistivity $1/\sigma$. It is a material property with typical values of $\mathrm {Pm} < 10^{-5}$ for liquid metals.

A background magnetic field can also affect non-axisymmetric instabilities. Progressively increasing the field strength, it initially suppresses existing hydrodynamic instabilities and subsequently leads to new magnetic instabilities (Hollerbach and Skinner 2001; Hollerbach 2009; Gissinger et al. 2011). A review of these can be found in Rüdiger et al. (2013). Magnetic fields can also affect inertial modes in the system and give rise to magneto-Coriolis modes (Schmitt et al. 2013). We survey below the hydromagnetic spherical Couette experiments with the setups shown in Fig. 8, except the 60 cm which has already been discussed above.

The first experiment we discuss is the 30-cm experiment from Maryland with an aspect ratio of 0.35 and liquid sodium as the working fluid with Hall probes used for diagnostics (Sisan et al. 2004). A coaxial magnetic field is applied using a pair of electromagnets, but unlike the 60-cm, the field is strong enough to cause the onset of magnetic instabilities. The experimental observations had some similarity to the magnetorotational instability (MRI), used to explain angular momentum transport in accretion disks (Balbus and Hawley 1998). Simulations later showed that these observations were likely to be magnetic instabilities of the Shercliff layers (Gissinger et al. 2011). Recent numerical work with magnetic Couette flow has indeed found evidence of MRI (Meduri et al. 2019), but under conditions that would have been inaccessible by the 30-cm experiment.

After the initial water experiments discussed in Sect. 4.1, the 3-meter experiment at Maryland was commissioned to use liquid sodium as the working fluid for conducting MHD experiments with the aim of obtaining self-consistent dynamo action. A magnetic field was applied using a single current-carrying coil around the equator of the outer sphere. Under weak magnetic field, the flow was still primarily hydrodynamic. The bi-stable torque state already observed in water experiments (Sect. 4.1) was observed in the induced magnetic field as well, with the low (high)-torque state leading to a high (low) $\varOmega$-effect—stretching of magnetic field lines by zonal flow to create strong azimuthal magnetic fields. At high magnetic field strengths, there was a reduction in zonal field amplification by the $\varOmega$-effect and short bursts of magnetic field appeared in the same sense as the applied field (Zimmerman et al. 2014). These short bursts might indicate a state close to the onset of dynamo action (Raynaud and Dormy 2013). Although numerical simulations have shown that spherical Couette flow can potentially drive and sustain self-consistent dynamos (Guervilly and Cardin 2010; Cao et al. 2012), none have been observed in experiments yet. While the 3-meter system has not shown self-sustaining dynamo action, substantial amplification of the imposed background field by almost one order of magnitude has been observed (Zimmerman et al. 2014).

The Derviche Tourneur sodium (DTS) experiment at ISTerre, Grenoble, France, has a diameter of 42 cm, aspect ratio of 0.35, and also uses liquid sodium as a working fluid. The background magnetic field is an axial dipole, being produced using permanent magnets encased in the inner sphere. The experiment is designed to operate in the *magnetostrophic* regime where the pressure gradient, the Coriolis acceleration, and the Lorentz force balance each other—a state expected in the outer core of the Earth. Flow diagnostics in the past were carried out using voltage probes, ultrasonic Doppler velocimetry, and giant magneto-resistance (GMR) sensors. Details can be found in Nataf et al. (2006), Schmitt et al. (2008), Brito et al. (2011), Schmitt et al. (2013). The setup was recently upgraded to the new DTS-$\varOmega$ which has embedded electronic hardware rotating with the outer sphere, capable of recording more than 200 data channels at up to 10 kHz (Tigrine et al. 2019). The experiments with DTS found evidence of super-rotating jets, but the observed flow speeds showed a departure from numerical predictions (Nataf et al. 2006; Dormy et al. 1998). The experiments also observed the presence of magneto-Coriolis modes which are oscillatory modes similar to inertial modes, but the Lorentz force participates as a restoring force as well (Schmitt et al. 2008, 2013). More recently, numerical and experimental studies of DTS-$\varOmega$ revealed different dynamic states based on inner sphere rotation rate—from a quasi-geostrophic base flow to large-scale structures on top of a turbulent background (Kaplan et al. 2018). The setup was also used to study torsional Alfvén waves thought to be present in the Earth’s outer core (Tigrine et al. 2019).

The HEDGEHOG experiment (Hydromagnetic Experiment with Differentially Gyrating sphEres HOlding GaInSn) is a magnetized spherical Couette experiment built recently at the Helmholtz-Zentrum Dresden-Rossendorf (HZDR) in Germany (Kasprzyk et al. 2017). This is a device in some ways similar to the 30-cm experiment, but instead of liquid sodium, HEDGEHOG uses GaInSn, an eutectic liquid metal alloy as the working fluid. The outer, 18-cm-diameter sphere is stationary, and the inner sphere can be 6 cm or 9 cm in diameter. The device is immersed in a homogeneous magnetic field provided by two external electromagnet coils. The flows explored by HEDGEHOG are quasi-laminar, and the applied magnetic field is relatively weak. Depending on the Hartmann number, different instabilities appear as successive Hopf bifurcations featuring a specific type of waves called quasi-periodic modulated rotating waves (MRW) (Garcia et al. 2021). These are expected on theoretical grounds given the symmetry characteristics of the spherical Couette system (Crawford and Knobloch 1991). Numerical simulations show that these quasi-periodic states may contain up to three and even four fundamental frequencies before transitioning to chaos. This is nothing less than a manifestation of the Newhouse–Ruelle–Takens (NRT) transition to chaos scenario (Garcia et al. 2020). Group-theoretical considerations such as these might prove relevant and useful in the analysis of other experiments and perhaps for the Earth’s interior as well.

When thermal effects are included, the shear due to spherical Couette system can have interesting effects on convective flow. Both flows together can lead to the formation of asymmetric convection cells. The linear stability space for the system is then bounded by two unstable regimes—one due to convection and another due to shear flow instabilities. Lastly, the heat transport efficiency (given by the Nusselt number) increases with differential rotation. More discussion on this problem can be found in Travnikov and Egbers (2020).

In conclusion, the spherical Couette system provides a relatively simple system to study a plethora of hydrodynamic and magnetohydrodynamic instabilities and phenomena that can occur in rotating spherical shells. This not only makes it yet another “Drosophila” of fluid dynamics alongside Taylor–Couette flow and Rayleigh–Bénard convection (van Gils et al. 2012), but one whose geometry is closer to the interiors of astrophysical objects, including the Earth. The 3-meter liquid sodium experiment is, as of this writing, being modified to include baffles on the inner sphere. These baffles have been designed in such a way that they enhance substantially the angular momentum communicated to the fluid by the inner sphere, thus generating a more pronounced $\varOmega$-effect. Hopefully, this will increase the chances to obtain dynamo action. There are also plenty of opportunities for progress using hydrodynamic experiments and numerical simulations, particularly related to the nature of the bi-stability phenomenon and its peculiar angular momentum characteristics, of which we understand very little. The recently discovered Rossby waves in the Sun (Löptien et al. 2018) constitute yet another potential application area for the spherical Couette system.

## Effects of Boundary Topography

Boundary topography in planetary liquid layers, including cores and subsurface oceans, exists on different length scales (see also Dehant 2022). First at the largest scales, we have already seen in Sects. 2 and 3 that planets have an ellipsoidal shape, resulting from their polar flattening superimposed to their mostly equatorial tidal distortions. Then, meso-scale topography may also develop at the core–mantle boundary (CMB) of a planet or at the top and bottom of a subsurface ocean. On Earth, CMB topography is mostly controlled by the dynamics of the Earth’s mantle, where it is assumed that uprising material and plumes drag the CMB upward into the mantle, while cold and dense material would deflect the CMB toward the core (Deschamps et al. 2018). Based on seismological observations and/or geodynamical considerations, several different models for the CMB topography have been proposed, with topography on various length scales but mostly confined to amplitudes smaller than 10 km (Morelli and Dziewonski 1987; Soldati et al. 2012; Sze and van der Hilst 2003; Tanaka 2010). Finally, it has been proposed that physical and chemical alteration of the crystalline mantle in direct contact with the hot core fluid may result in roughness at the top of the core. This would be confined to length scales as small as tens of centimeters to meters, i.e., on the order of the thickness of the viscous boundary layer (Narteau et al. 2001).

The wide range of topography wave length and amplitude gives rise to various physical interaction mechanisms between the solid and liquid layer, potentially leading to exchange of angular momentum and energy dissipation, that may be reflected in the rotational parameters of the planet. While large-scale topography, such as polar flattening or tidal deformations, essentially affects the structure, frequency, and global stability of inertial modes, smaller scales are expected to have a more local effect that might propagate into the bulk in the form of inertial waves and Rossby waves.

Investigating the effects of topography on the dynamics of fluid planetary cores requires three key ingredients: (1) strong background rotation, (2) a non-spherical surface, and (3) a mechanism to drive fluid motions. It is especially the second of these requirements that makes such studies challenging, even for the most advanced numerical solvers at present. While efficient spectral methods can be used in spheres and spherical shells to approach the parameters of rapidly rotating regimes, they fail at resolving more complex geometries for which one has to rely on computationally more expensive (e.g., finite element) simulations. However, the latter are not well suited to approach the low Ekman number regimes characteristic of planetary settings.

In contrast, laboratory experiments are well suited to investigate the effects of topography on planetary core dynamics as they allow to investigate a wide range of topography while still achieving comparable or smaller Ekman numbers than modern numerical methods. A typical experimental setup consists of a container of the desired shape, usually filled with water as working fluid, which is mounted on a turntable to mimic the planetary rotation. Large-scale topography studies are performed in spheroids or ellipsoids (see Figs. 3 and 4), while meso- and small-scale topographies are more easily implemented in a cylindrical geometry. Main characteristics of the various setups described in this section are listed in Table 4. It should be noted that in all the aforementioned experiments, the amplitude of the topography is highly exaggerated with respect to planetary cores, to compensate for the increased influence of dissipation (i.e., larger Ekman numbers in experiments). For example, the typical oblateness of a spheroid in experiments is on the order of 1/10, one to two orders of magnitude larger than in planetary settings. Geophysically relevant flow can be generated by introducing a temperature gradient to mimic convection (Westerburg and Busse 2003), or by mechanical forcings.

**Table 4:** Parameters of selected studies related to effects of core–mantle boundary topography

Reference	Topography scale	Geometry	Forcing
Charles (2018)	Large scale	Triaxial ellipsoid	Libration
Westerburg and Busse (2003), Jaletzky and Busse (2000)	Meso scale	Cylindrical annulus	Thermal
Burmann and Noir (2018)	Meso/small scale	Cylinder	Spin-up

**Fig. 9:**
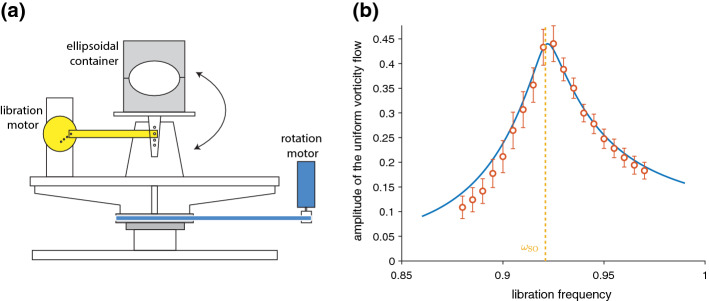
Experimental study of large-scale topography: flows driven by latitudinal librations in a triaxial ellipsoid (Charles 2018). **a** Experimental setup. **b** Amplitude of the uniform vorticity flow as a function of the libration frequency (red circles) showing resonance in agreement with the theoretical prediction (blue curve). The yellow line marks the predicted frequency of the spin-over mode from Vantieghem et al. (2015)

The largest-scale deformations of planets are long known, and their influence on the fluid flows in planetary cores has been the subject of theoretical considerations since the end of the nineteenth century (Hough 1895; Sloudsky 1895; Poincaré 1910). Beyond the already discussed flows driven by precession and libration (see Sects. 2 and 3), nutations and latitudinal librations of the mantle also generate flows through coupling with large-scale topography. As for precession (see Sect. 2), they force the liquid core to rotate along a tilted axis, which can be decomposed in an order one axial rotation and a small rotation along an equatorial axis. The latter is referred to as the spin-over mode, the simplest (linear in the spatial coordinates) inertial mode of a rotating fluid cavity. The eigenfrequency of this mode is determined by the geometry of the cavity: For a sphere, the mode has the same frequency as the rotation, whereas in an oblate spheroid its eigenfrequency is smaller, and in a prolate spheroid it is larger than the rotation frequency. All of the aforementioned mechanical forcings will excite this mode, potentially leading to a resonance when the forcing frequency is equal to the eigenfrequency of the mode. Detecting this resonance thus becomes a way to probe the first-order departure from an ideal, purely spherical CMB.

An example of the pseudo-resonance occurring in a librating ellipsoid has been reported in Charles (2018), who experimentally investigated the flows driven by libration in latitude. The experiment consists of an ellipsoidal container characterized by its three semi-axes of length $a\ne b \ne c$, which is mounted on a turntable. The ellipsoidal container is harmonically oscillating around a second axis, which is inclined by $90^\circ$ with respect to the rotation axis (Fig. 9a). Spatially resolved flow measurements, along several chords inside the fluid, are performed with ultrasonic Doppler velocimetry (UDV), and the positioning of the measurement profiles allows a time-resolved determination of all three components of the spin-over mode. It can be shown that no inviscid spin-over mode can be excited in an ellipsoid which is axisymmetric along the libration axis (Vantieghem et al. 2015). In a triaxial ellipsoid, however, a spin-over mode can grow with any orientation of the container. Following Vantieghem et al. (2015), a theoretical prediction of the three components of the spin-over mode is derived as a function of the ellipticity and oscillation frequency, and a comparison with the experimental UDV data yields a very good agreement (see Fig. 9b), thus validating the theoretical model of the spin-over mode in a triaxial ellipsoid.

The effect of meso-scale topography has been widely studied in atmospheric and oceanic contexts with a clear emphasis on stratified fluids, but only few studies so far have considered meso-scale topography with a focus on planetary core dynamics, most of which are either theoretical or numerical. Using a quasi-geostrophic approximation, Calkins et al. (2012) investigated numerically the interplay between a meridional ridge and thermally driven core convection. They report the excitation of thermal Rossby waves with a wavelength scaling as $Ro^{1/2}$, demonstrating that topography can transfer energy from large-scale zonal flows to small-scale flow structures. Such waves have also been reported in theoretical works (e.g., Bell and Soward 1996; Herrmann and Busse 1998) and in experiments by Westerburg and Busse (2003). The latter investigated the effects of sinusoidal-shaped end-walls on the thermal convection in a rotating cylindrical annulus. Their experimental apparatus is a modified version of the one described in detail by Jaletzky and Busse (2000). Convection is driven in a rapidly rotating cylindrical annulus, filled with either nitrogen or water, by applying a temperature gradient between the cool inner and warm outer cylinder wall. The rotation rate is chosen in such a way that centrifugal forces dominate over gravity. To detect the thermal Rossby waves, thermistors are attached to the inner wall of the cavity. A comparison between theoretical and/or numerical predictions yields a semiquantitative agreement, and it is concluded that thermal Rossby waves may significantly influence the core convection close to the tangent cylinder.

**Fig. 10:**
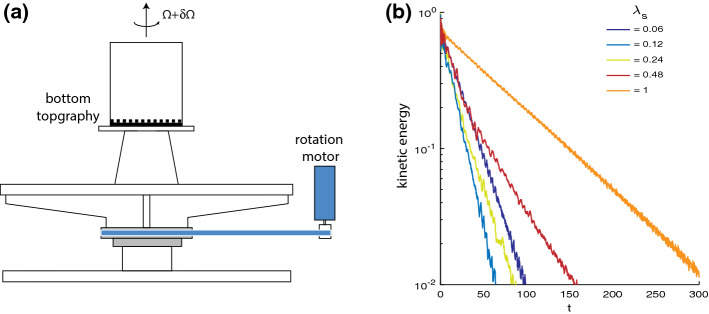
Investigation of meso-scale topography. Spin-up experiments in a cylinder with bottom topography (Burmann and Noir 2018): **a** experimental setup; **b** kinetic energy as a function of time. The decay of the kinetic energy depends on the length scale of the bottom topography $\lambda _s$ (ratio between the block size and the radius of the cylindrical container). Topography results in a faster decay of the kinetic energy (all curves where $\lambda _s\ne 1$), i.e., enhances the dissipation in the system

Motivated by the challenge to better characterize the transport of energy and angular momentum from the topography to the fluid flow, Burmann and Noir (2018) carried out a study of the spin-up of a rapidly rotating fluid with bottom topography. The experimental setup consists of a rotating straight circular cylinder with a chessboard like arrangement of rectangular blocks at the bottom (Fig. 10a). Small differential fluid motions with respect to the topography are created by an abrupt small increase in the rotation rate, the so-called spin-up (Greenspan and Howard 1963). The resulting flow is diagnosed in three horizontal planes using two-dimensional particle image velocimetry. Using the decay rate of the kinetic energy as a proxy for the coupling between the fluid and the container, it is found that topography enhances the exchange of angular momentum and energy dissipation (Fig. 10b), an effect that is maximized for a specific horizontal length scale of the blocks. The authors show that shortly after the abrupt change of rotation, nonlinear interacting inertial waves fill the cylindrical volume carrying momentum away from the bottom topography, eventually forming columnar structures with a horizontal length scale commensurate with the length scale of the bottom topography. To translate these results to a geophysical context, further studies are required in particular at lower Ekman number.

To conclude, we note that there remains the need for more, especially experimental, work to obtain a deeper understanding of topographic effects in planetary cores. While there is a good understanding from the numerous work on the large-scale deformations (spheroids and ellipsoids) under various forcing, the sparse literature on medium- and small-scale topography should motivate for further research in this direction. Such studies should include a more realistic representation of the CMB topography as well as a focus on smallest-scale topography, which has been overlooked by experimentalists with a focus on planetary core dynamics.

## Conclusions

The dynamics of planetary interiors is intrinsically an interdisciplinary research area. We are convinced that it should also be tackled by a collaborative, multi-method approach. Indeed, it is obviously out of reach for any model, even with the most powerful present-day supercomputers, to include simultaneously all the physics and timescales involved in, for example, a core flow history since its formation. The classical approach decomposes the global problem into well-defined restricted models addressing specific points: A systematic exploration of the parameter space, coupled with an in-depth understanding of the underlying physics, then allows deriving generic scaling laws that are extrapolated toward planetary scales and challenged against available data. Against this backdrop, this review article is aimed at illustrating the value of laboratory experiments, that allow in particular to reach the most demanding regimes for long data acquisition. The drawbacks are of course the difficulty in data acquisition, as well as the limitations of accessible geometries and physics compared to simulations: e.g., physical properties of conducting fluids available in the laboratory still render the realization of experimental dynamos extremely challenging. Laboratory experiments have resulted in significant progress on understanding the turbulent dynamics in rotating systems of relevance to planetary flows. And as demonstrated by the few examples here, adequately combining the advantages of simulations with dedicated experiments will allow us to keep progressing in our understanding of planetary interiors and to take the most of the increasing flux of data and knowledge from observational missions, on Earth, in our solar system and beyond.

Beyond these general thoughts, we conclude this review by listing, in our view, the three main upcoming challenges for experimental planetary fluid dynamics. As briefly discussed in Sect. 3 in the context of libration forcing, the state of turbulence in planetary relevant regimes remains controversial. Up to now, following the many studies of convection in fast rotating spherical shells, the efforts of the community have mainly focused on characterizing strong, geostrophic turbulence (Schaeffer et al. 2017). But it might turn out that the relevant regime is actually weak, wave turbulence (Le Reun et al. 2019). Our representation of flow scales and organization, hence our understanding of their repercussion on planetary dynamics including dissipation and magnetic field generation, would then have to be fully refounded. This discussion is not restricted to libration forcing: It is actually relevant for any rotating flow, excited by any type of mechanical forcing. The competition between the two different turbulent regimes might explain some of the open questions persisting in precession (Sect. 2) as well as in spherical Couette flows (Sect. 4). The second challenge for experimental studies will be to combine the various types of forcings. Indeed, while we have up to now made great progress in understanding each type of mechanical forcing independently, it turns out that several, if not all, take place simultaneously in real planets. The question also remains to understand how they couple with buoyancy effects. For instance, the Earth’s core is both convecting and precessing, with a CMB topography at both small and large scales; its inner core may also rotate differentially. The Moon’s liquid core has been stably stratified for a long time and has been simultaneously shaken by both precession and libration with greater amplitudes in the past; its boundaries also exhibit topography at both small and large scales. The question remains to understand whether simultaneous buoyancy and/or mechanical forcings will simply superimpose their effects, will cancel each other, or will lead to new, interesting nonlinear couplings. This has only been studied in few studies in cylindrical geometry up to now (Lavorel and Le Bars 2010; Guimbard et al. 2010). Finally, the third great experimental challenge we would like to highlight here is to obtain, at last, a fluid dynamo in the laboratory. Indeed, the three successful experimental dynamos obtained up to now were always very constrained, either in the flow organization (Gailitis et al. 2001; Stieglitz and Müller 2001) or in their boundary conditions (Berhanu et al. 2010). Two dedicated large-scale setups are presently in construction in Dresden (Giesecke et al. 2018), or under amelioration for the 3-meter liquid sodium experiment in Maryland, to finally reach the dynamo threshold in fully turbulent flows. Exciting results are to be expected in the near future.
